# Role of Exosomal Non-Coding RNAs in Bone-Related Diseases

**DOI:** 10.3389/fcell.2021.811666

**Published:** 2021-12-23

**Authors:** Hang Li, Qiyue Zheng, Xinyan Xie, Jiaojiao Wang, Haihong Zhu, Haoye Hu, Hao He, Qiong Lu

**Affiliations:** ^1^ Department of Pharmacy, The Second Xiangya Hospital, Central South University, Changsha, China; ^2^ Institute of Clinical Pharmacy, Central South University, Changsha, China; ^3^ College of Traditional Chinese Medicine, Guangzhou University of Chinese Medicine, Guangzhou, China; ^4^ Department of Medical Genetics, The Second Xiangya Hospital, Central South University, Changsha, China; ^5^ Department of Vascular Surgery, The Second Xiangya Hospital of Central South University, Changsha, China

**Keywords:** bone-related diseases, exosomes, non-coding RNAs, osteoporosis, osteoarthritis

## Abstract

Bone-related diseases seriously affect the lives of patients and carry a heavy economic burden on society. Treatment methods cannot meet the diverse clinical needs of affected patients. Exosomes participate in the occurrence and development of many diseases through intercellular communication, including bone-related diseases. Studies have shown that exosomes can take-up and “package” non-coding RNAs and “deliver” them to recipient cells, thereby regulating the function of recipient cells. The exosomal non-coding RNAs secreted by osteoblasts, osteoclasts, chondrocytes, and other cells are involved in the regulation of bone-related diseases by inhibiting osteoclasts, enhancing chondrocyte activity and promoting angiogenesis. Here, we summarize the role and therapeutic potential of exosomal non-coding RNAs in the bone-related diseases osteoporosis, osteoarthritis, and bone-fracture healing, and discuss the clinical application of exosomes in patients with bone-related diseases.

## Introduction

Bone is one of the most complex tissues in mammals. It undergoes continuous shaping, remodeling and repair throughout its life cycle to protect vital organs and provide rigid support for the entire body (Long and Ornitz, 2013; Riddle and Clemens, 2017). Bone-related diseases are global health problems that seriously affect the quality of life of patients, and include osteoporosis (OP), osteoarthritis (OA), and fracture, etc. (Manolagas, 2013; Liu et al., 2015b). With the aging of the population becoming serious, it is estimated that the global OP and OA patients have reached 200 million and 250 million, respectively (Vos et al., 2012; Weaver et al., 2016).

Bone homeostasis is maintained in two main parts: osteoblast-mediated bone formation and osteoclast-mediated bone resorption. In addition, bone-marrow adipocytes, stromal cells, and osteocytes in the bone microenvironment contribute to the maintenance of bone homeostasis (Lems and Raterman, 2017; Chawalitpong et al., 2018; Zhao et al., 2018). Bone homeostasis also gradually declines with aging during bone remodeling. Enhanced bone resorption or weakened bone formation can lead to disruption of bone homeostasis, which can result in severe bone loss and osteoporotic fractures in older and postmenopausal women (Matsuo and Irie, 2008; Cao, 2011). The moieties involved in osteoblast-mediated bone formation and osteoclast-mediated bone resorption are signaling proteins, and they maintain bone homeostasis. To ensure the integrity and versatility of bones, bone remodeling occurs throughout life (Zhao et al., 2006; Suchacki et al., 2017). It has also been proposed that osteocytes have essential roles in bone remodeling because they affect the activities of osteoblasts and osteoclasts (Tatsumi et al., 2007).

Exosomes are membrane-bound extracellular vesicles produced in the endosomal compartment of most eukaryotic cells. Exosomes have been shown to be important vehicles for communication between bone cells that maintain bone homeostasis (Kalluri, 2016). Recent studies have demonstrated that exosomes secreted by bone marrow mesenchymal stem cells (BMSCs), osteoclasts, and osteoblasts are involved in the regulation of bone metabolism. Osteoblast activity is inhibited by exosomes secreted by osteoclasts, thereby inhibiting the bone formation-activity of osteoblasts (Li et al., 2016; Sun et al., 2016). However, the promotion of osteoblast differentiation is regulated through exosomes secreted by osteoblasts and BMSCs (Cui et al., 2016; Xu and Wang, 2017).

**FIGURE 1:**
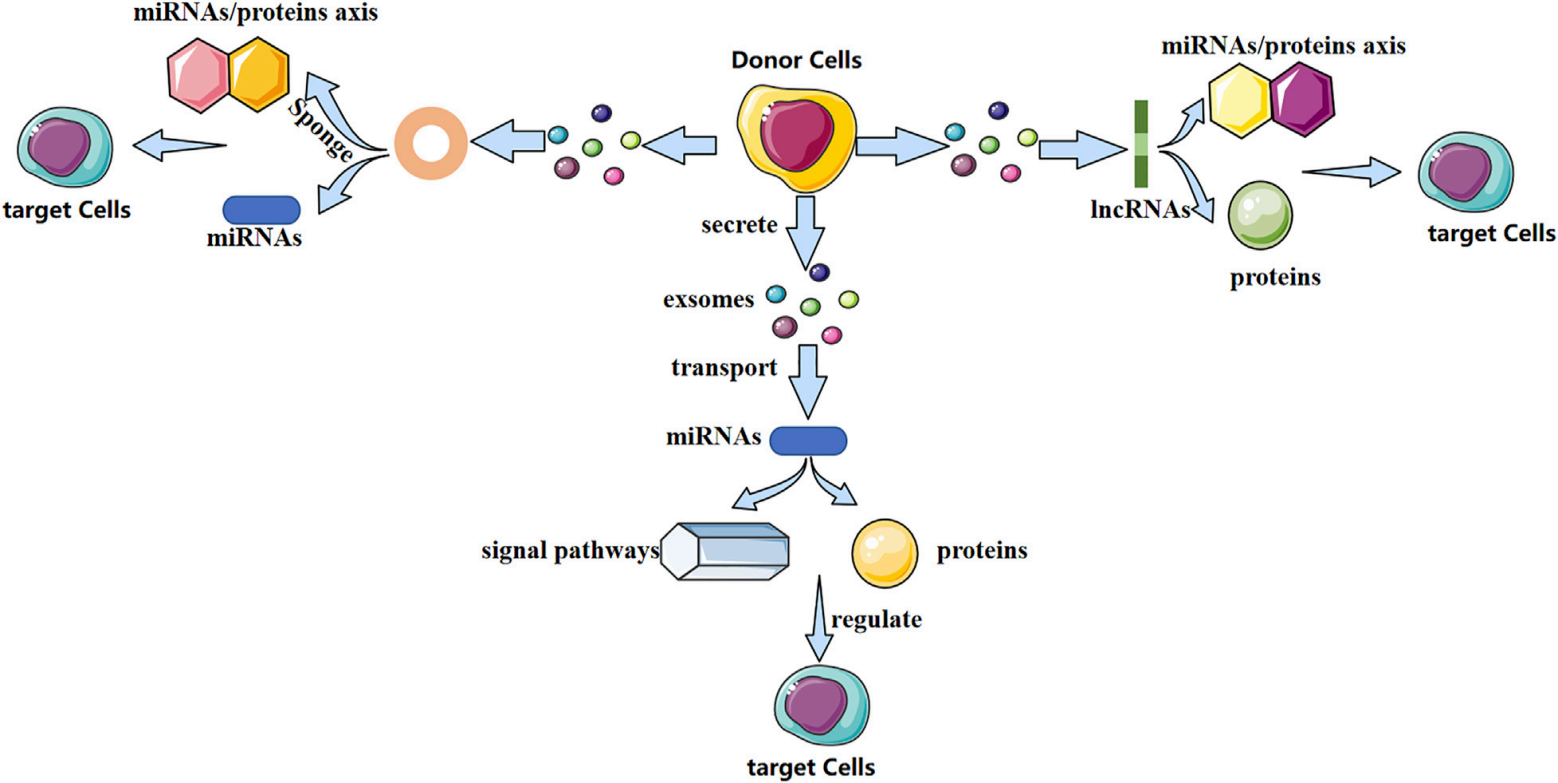
Possible mechanism of action of three exosomal non-coding RNAs (schematic). Different exosomal non-coding RNAs (miRNA, lncRNA, and circRNA) regulate the fate and differentiation of target cells through different mechanisms, thereby affecting disease progression.

Exosomes contain various biologically active molecules, which can be delivered to target cells through ligand–receptor interactions, endocytosis, direct membrane fusion, or conduction of signaling pathways (Kalamvoki et al., 2014). In particular, non-coding RNAs (ncRNAs) are present in exosomes. Research of exosomal ncRNAs in breast cancer (Naseri et al., 2018), neurologic diseases (Xin et al., 2012), nephropathy (Wang et al., 2019), and other diseases has attracted considerable attention. Different exosomal ncRNAs have different mechanisms in bone-related diseases (Figure 1). They play a vital part in bone remodeling and have an indispensable role in bone-related diseases.

The purpose of this review is to demonstrate the role of exosomal non-coding RNAs in bone-related diseases and discuss its potential clinical applications. With a view to providing new research directions and ideas for bone-related diseases in the aging process.

## Functions and Contents of Exosomes

Exosomes were discovered ∼40 years ago. Their diameter is, in general, between 40 and 150 nm (Pan and Johnstone, 1983; Zhou et al., 2017). Formation of early endosomes is by invagination. Then, under the regulation of endosomal transport complexes and some related proteins, these early endosomes “sprout” and form multivesicular bodies. The latter can release vesicles after fusion with the plasma membrane, and exosomes are formed (Simons and Raposo, 2009; Soekmadji et al., 2013).

An increasing number of cells have been shown to secrete exosomes: MSCs, lymphocytes and macrophages (van Niel et al., 2001; Bourdonnay et al., 2015; Guay et al., 2019). Exosomes mediate intracellular communication through biologically active molecules such as, proteins, long non-coding (lnc)RNAs and microRNAs (miRNAs) that they contain (Thery et al., 2002; Kowal et al., 2014). Exosomes have been studied extensively in the last decade due to their multiple functions in various physiological processes and diseases. Exosomes can participate directly in signal transmission between cells, which is one of the most important factors in paracrine regulation (Dai et al., 2019).

**FIGURE 2:**
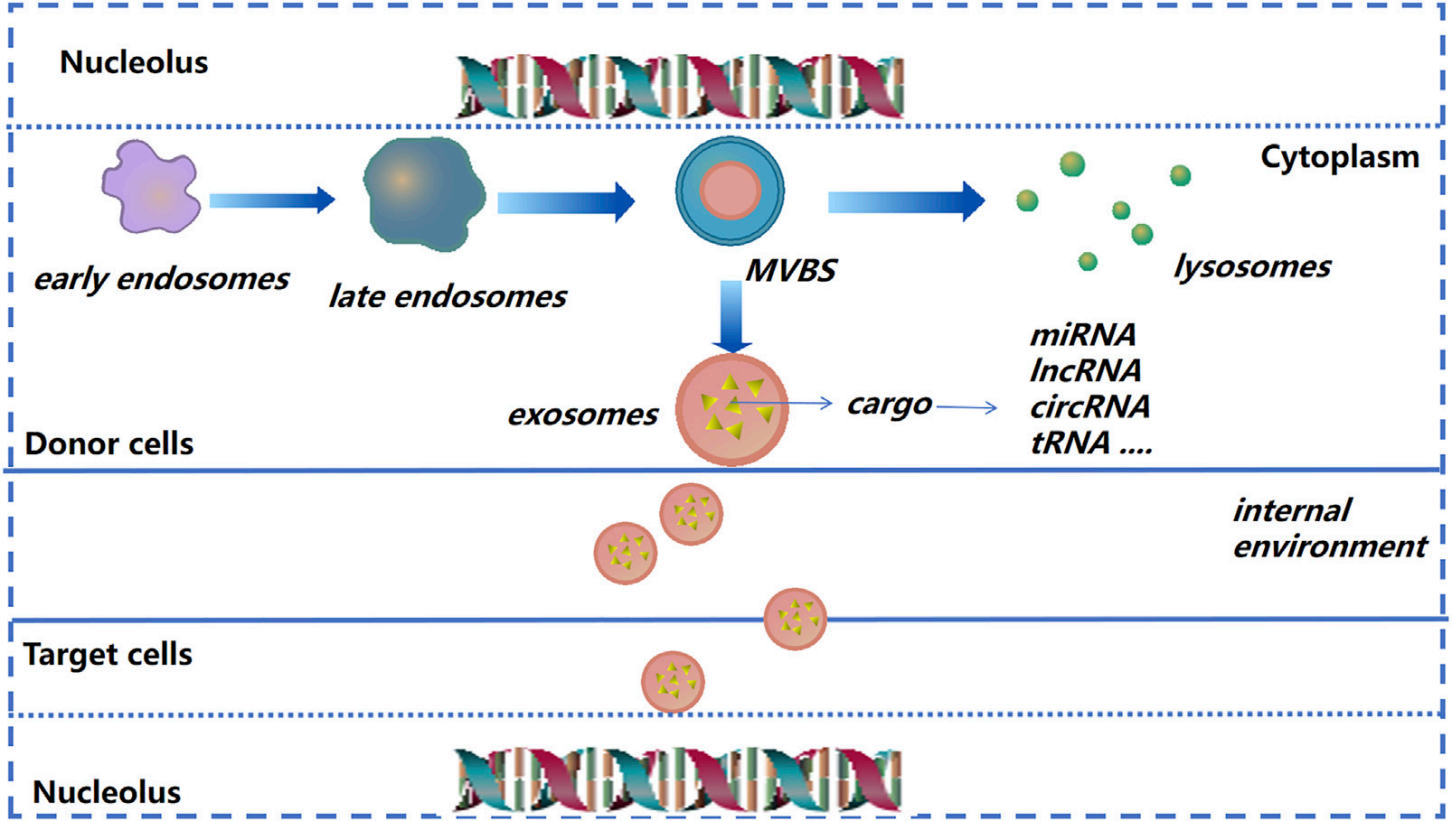
Exosome formation and cargo release (schematic). As the endocardium of the early endosome forms a vesicle into the inner bud, an exosome begins to form and then transform into multivesicular bodies. The latter can release vesicles after fusion with the plasma membrane, and are then known as “exosomes.”

After donor cells secrete exosomes into the extracellular matrix (ECM), exosomes will be recognized and internalized by recipient cells; then exosomes release their contents into recipient cells to regulate cell function (Kalluri and LeBleu, 2020) (Figure 2). Due to their small size, stable structure, low toxicity, and other characteristics, exosomes are being employed increasingly as “nano-medicine carriers” for tissue regeneration and disease treatment (El-Andaloussi et al., 2012; Batrakova and Kim, 2015; Yang et al., 2019a). In general, the separation method is selected based on the experimental principle and source of exosomes. Ultracentrifugation is used as the “gold standard” for separation and purification of exosomes, among which density gradient ultracentrifugation is the best method. The advantages are simple pretreatment, non-requirement of specialist knowledge, and economic affordability (Li et al., 2017). The exosomes prepared by size-exclusion chromatography have high purity, which can preserve the integrity of vesicles and prevent exosome aggregation (Patel et al., 2019). Immunoaffinity isolation, field-flow fractionation, and precipitation are also used in the isolation and extraction of exosomes (Kang et al., 2017; Gurunathan et al., 2019; Zhang and Lyden, 2019).

## Classification and Function of ncRNAs

Mammalian cellular RNAs have been studied for decades (Morris and Mattick, 2014). Advances in sequencing technology have led to the discovery of many ncRNAs. The latter include circular RNAs (circRNAs), miRNAs, lncRNAs, and transfer RNAs (tRNAs) (Sharp, 2009; Ling et al., 2013; St Laurent et al., 2015; Ebbesen et al., 2016). According to the difference in length, ncRNAs can be divided into small ncRNAs and lncRNAs (Knowling and Morris, 2011). Only ∼2% of the human genome is transcribed into proteins, most of the remainder are transcribed into ncRNAs of different sizes and functions, so a large class of RNAs does not code for proteins (Iyer et al., 2015; Gupta and Thum, 2016). ncRNAs participate in several biological processes, including regulating gene expression at the transcriptional level and directing genome rearrangement or DNA synthesis (Cech and Steitz, 2014). The lncRNAs associated with chromatin modification have been described recently. Small RNAs transcribed from promoter regions and bidirectional promoters produce ncRNAs of different sizes (Khalil et al., 2009; Neil et al., 2009; Taft et al., 2009). Under certain conditions, miRNAs expression can be used to accurately identify the origin of poorly differentiated tumors compared with that using protein-encoded messenger (m)RNAs, so ncRNAs could be “ideal” diagnostic markers (Rosenfeld et al., 2008). Besides, studies have shown that ∼200 miRNAs have been characterized sufficiently to enable classification of cancer types. Also, spectral analyses of miRNAs seem to overcome some of the difficulties of early detection associated with colon cancer and other occult cancers (Lu et al., 2005; Aslam et al., 2009; Cortez and Calin, 2009). Such large numbers of ncRNAs with diverse mechanisms constitute a huge and efficient gene-regulatory network, which is involved in many physiological and pathological processes (Ulitsky and Bartel, 2013; Zhang et al., 2019b; Wu et al., 2020).

## Roles of ncRNAs in Bone-Related Diseases

### Roles of Exosomal miRNAs in Bone-Related Diseases

**FIGURE 3:**
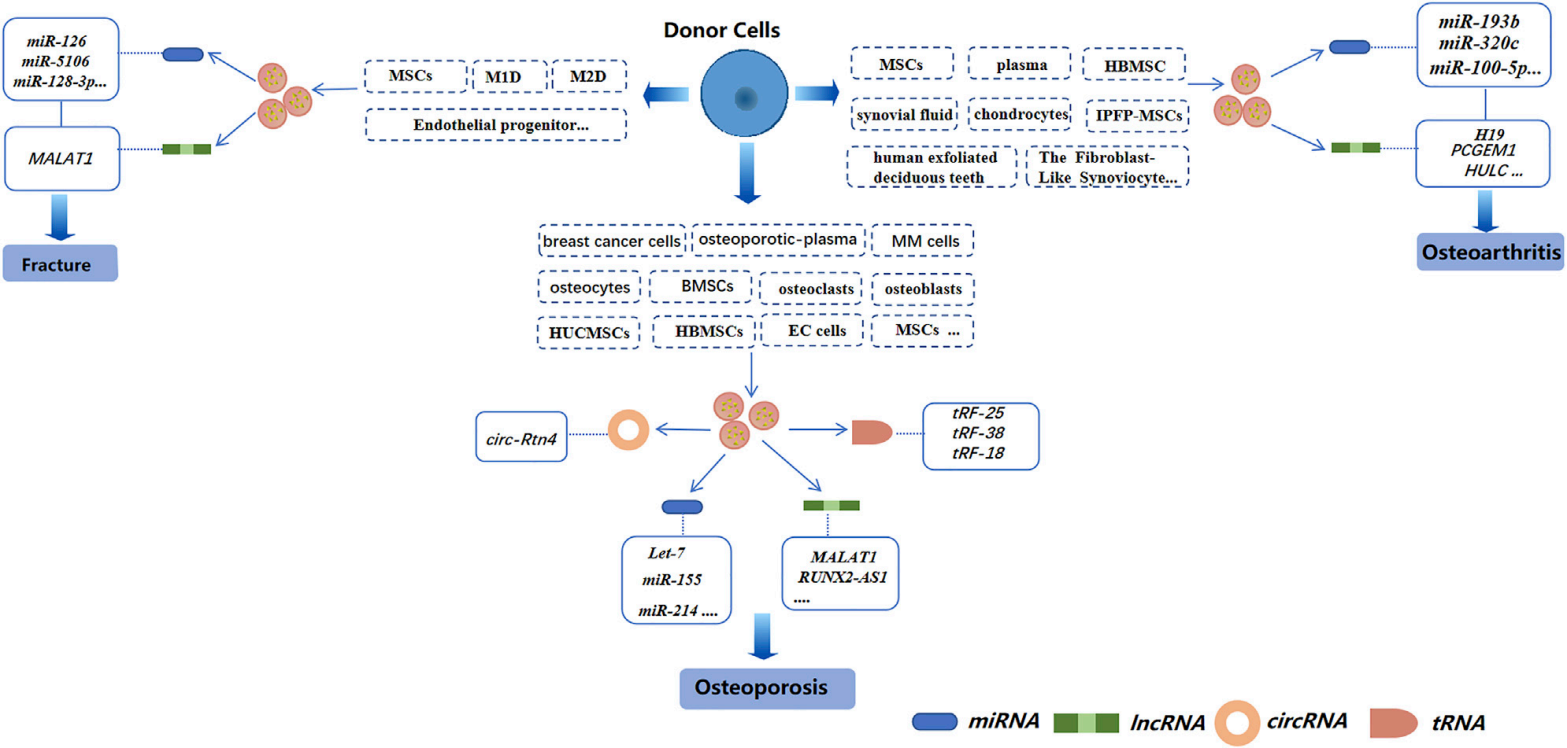
Exosomal non-coding RNAs derived from different cells in three bone-related diseases (schematic). Different sources of exosomes, such as BMSCs, serum, and cancer-cell sources, have different effects on osteoporosis, osteoarthritis, and bone fractures by regulating different exosomal non-coding RNAs.

miRNAs are small coding RNAs derived from a hairpin or double-stranded RNA precursor of length ∼22 nucleotides. miRNAs are generated from introns and exons of protein-encoded and non-coding transcripts by RNA polymerase II (Lee et al., 2003; Landthaler et al., 2004; Mattick and Makunin, 2005). miRNAs are involved in regulating the proliferation, differentiation, and apoptosis of cells, as well as embryonic development (Ambros, 2003; Giraldez et al., 2005; Hatfield et al., 2005; Naguibneva et al., 2006; Plasterk, 2006; Lee et al., 2015). Several studies have demonstrated miRNAs to be involved in the regulation of osteoclasts and osteoblasts. For example, inhibiting miR-31 expression blunts osteoclast formation and bone resorption (Mizoguchi et al., 2013). Besides, miR-140-3p can regulate osteoblast differentiation by targeting the transforming growth factor (TGF)β3 signaling pathway (Fushimi et al., 2018). Exosomal miRNAs from different cells have also been studied extensively in bone-related diseases (Figure 3).

#### Roles of Exosomal miRNAs in OP

OP is characterized by fragile bones and an increased risk of fracture. OP may be a systemic deterioration of bone mass and bone microstructure due to bone metabolic imbalance (Rachner et al., 2011). OP can lead to a decline in the quality of life of patients, but also bring about a huge economic burden to society (Curtis et al., 2016). miRNAs have been shown to play an important part in OP (Tang et al., 2017) (Table.1).

**TABLE 1:** The role of exosomal non-coding RNAs in osteoporosis.

Origin of exosomes	Exosomes cargo	Pathway	ncRNA expression	Mechanism	References
Osteoclasts	miR-214	EphrinA2/EphA2	high	inhibited the function of osteoblasts	Sun et al. (2016)
Osteoclasts	miR-214-3p	N/A	high	inhibited osteoblast bone formation	Li et al. (2016)
Osteoblasts	miR-30d-5p	RUNX2	high	inhibit osteoblast differentiation	Li et al. (2008); Zhang et al. (2011)
	miR-133b-3p				
Osteoblasts	miR-140-3p	BMP2	high	inhibited the formation of osteoblasts	Hwang et al. (2014)
osteoblast precursors/mineralized osteoblasts	Let-7	HMGA2	high	promoted osteogenesis	Egea et al. (2012); Wei et al. (2014)
	AXIN2				
Osteoblast	miR-503-3p	RANK	N/A	preventd osteoclast differentiation	Chen et al. (2014)
mineralized osteoblasts	miR-667-3p	AXIN1	high	promoted the osteogenic differentiation of osteoblast precursors	Cui et al. (2016)
	miR-6769b-5p	β-catenin			
	miR-7044-5				
	pmiR-7668-3p miR-874-3p				
HBMSCs	miR-199b	N/A	High/low	increased/decreased significantly during the early stage of osteogenic differentiation of HBMSCs	Xu et al. (2014)
	miR-218				
	miR-148a				
	miR-135b/miR-221				
Osteocytes	miR-218	Wnt signaling	low	inhibited osteoblast differentiation	Qin et al. (2017)
BMSCs	miR-31a-5p	N/A	High	promoting osteoclastogenesis and bone resorption	Xu et al. (2018)
BMSCs	miR-151-5p	N/A	N/A	promoting osteogenic differentiation and saving bone reduction	Chen et al. (2017)
MSCs	miR-21	SMAD7	High	Inhibition of osteogenic gene expression	Jiang et al. (2018)
HUCMSCs	miR-1263	Mob1	High	inhibiting BMSCs apoptosis and preventing disuse osteoporosis in rats	Yang et al. (2020a)
breast cancer cells	miR-20a-5p	SRCIN1	N/A	Promoting the proliferation and differentiation of osteoclasts	Guo et al. (2019)
vascular endothelial cells	miR-155	N/A	High	inhibited osteoclast induction	Song et al. (2019)
HBMSCs exosome mimetics	miR-29a	Noggin	High	promoted osteogenesis	Fan et al. (2020)
BMSCs	miR-186	Hippo signaling pathway	High	promote osteogenesis	Li et al. (2021)
BMSCs	LncRNA MALAT1	MIR-34c/SATB2 axis	N/A	promoted osteoblast activity/enhanced the activity of osteoblasts in osteoporotic mice	Yang et al. (2019b)
MM cells	lncRNA RUNX2-AS1	RUNX2	N/A	inhibiting the osteogenicity of MSCs	Li et al. (2018)
osteoclasts	NONMMUT000375.2	genes related to osteoclast	N/A	repressed the osteogenic differentiation of MC3T3-E1 cells	Xu et al. (2020a)
	NONMMUT071578				
BMSCs	lncRNA H19	Angpt1/Tie2-NO	High	promoted osteogenesis and angiogenesis through mediating Angpt1/Tie2-NO signaling	Behera et al. (2021)
circ-Rtn4 modified BMSCs	circ-Rtn4	miR-146a	N/A	reduced the cytotoxicity and apoptosis of MC3T3-E1 cells induced by TNF-α	Cao et al. (2020)
serum samples	hsa_circ_0006859	miR-431-5p	High	Hsa_circ_0006859 suppressed osteoblastic differentiation and promoted adipogenic differentiation of hBMSCs.	Zhi et al. (2021)
osteoporotic plasma exosomes	tRF-25 tRF-38 tRF-18	N/A	High	had good accuracy in the diagnosis of osteoporosis	Zhang et al. (2018b)

The pathological process of OP is caused mainly by an imbalance between osteogenic differentiation and osteoclast differentiation. Recent studies have demonstrated that such imbalance can be regulated by exosomal ncRNAs. For instance, Sun and co-workers showed that the exosomes secreted by osteoclasts contain miR-214, which was transferred to osteoblasts through *Ephrin-A2/Eph-A2* recognition and inhibited osteoblast function (Sun et al., 2016). Li et al. (2016) showed the function of exosomal miR-214-3p in the communication between osteoclasts and osteoblasts. miR-214-3p can transfer from osteoclasts to osteoblasts and inhibit osteoblastic bone formation. Importantly, targeted inhibition of exosomal miR-214-3p in osteoclasts can reverse the inhibition of osteoblast activity and promote bone formation, which may be a potential treatment for bone loss. Studies have indicated that miR-30d-5p and miR-133b-3p can inhibit osteoblast differentiation by targeting the Runt-related transcription factor 2 (RUNX2) gene, and miR-30d-5p and miR-133b-3p show high expression in osteoblast-derived exosomes (Li et al., 2008; Zhang et al., 2011). Hwang et al. (2014) revealed that miR-140-3p had high expression in osteoblast exosomes. miR-140-3p inhibited the formation of osteoblasts by blunting expression of the bone morphogenetic protein 2 (BMP2) gene. Let-7 was identified in the osteoblast precursors and exosomes of mineralized osteoblasts, which promoted osteogenesis by regulating the high mobility AT-hook 2 gene and axis-like protein (AXIN)2 gene (Egea et al., 2012; Wei et al., 2014). Chen et al. (Chen et al., 2014) identified miR-503-3p in osteoblast-derived exosomes. They demonstrated that miR-503-3p could prevent osteoclast differentiation by inhibiting expression of the receptor activator of nuclear factor-kappa B (RANK) gene. miR-667-3p, miR-6769b-5p, miR-7044-5p, miR-7668-3p, and miR-874-3p which show high expression in the exosomes secreted by mineralized osteoblasts, can promote the osteogenic differentiation of osteoblast precursors by inhibiting *AXIN1* expression and enhancing *β*-catenin expression (Cui et al., 2016). These observations indicate that exosomal miRNAs from osteocytic cells can change the differentiation trend of osteoblasts and clasts, thereby inhibiting or promoting bone formation.

Xu et al. (2014) showed that expression of miR-199b, miR-218, miR-148a and miR-135b in exosomes increased significantly and miR-221 expression was decreased during the early stage of osteogenic differentiation of human bone marrow mesenchymal stem cells (hBMSCs). Besides, miR-199b, miR-218, miR-135b, and miR-148a have been suggested to be regulators in osteoblast differentiation in HBMSCs (Schaap-Oziemlak et al., 2010; Hassan et al., 2012; Cheng et al., 2013; Lauvrak et al., 2013; Xu et al., 2013). Qin et al. (2017) established that, after myostatin treatment, miR-218 expression in osteocyte-derived exosomes was downregulated, which could integrate into osteoblastic cells and inhibit osteoblast differentiation by downregulating the wingless type (Wnt) signaling pathway. Several key miRNAs related to osteogenesis (miR-34a, miR-27a, and miR-22) and adipogenesis (miR-143 and miR-375) have been detected in osteoblasts and adipocyte-derived exosomes, respectively (Narayanan et al., 2018). To study how exosomes promote osteogenic differentiation, Qin et al. (2016) detected miRNAs in exosomes, and found miR-27a, miR-206a, and miR-196a to have high expression. Furthermore, miR-196a had the greatest functional potential. Thus, targeting such exosomal miRNAs may be an ideal treatment strategy for patients with OP.

Rong et al. (Xu et al., 2018) demonstrated that miR-31a-5p could promote osteoclastogenesis and bone resorption, and that its expression in the exosomes of BMSCs of old rats was significantly higher than that in young rats. In addition, the differentiation and function of osteoclasts could be inhibited by blunting expression of miR-31a-5p in exosomes. miR-151-5p from the exosomes of exogenous BMSCs could be spliced into endogenous BMSCs to promote osteogenic differentiation. Bone reduction can be saved by injection of exosomal miR-151-5p *in vivo* (Chen et al., 2017). Jiang et al. (2018) showed that miR-21 expression in MSC-derived exosomes extracted from healthy adults was significantly lower than that of miR-21 in MSC-derived exosomes extracted from patients with OP.

**FIGURE 4:**
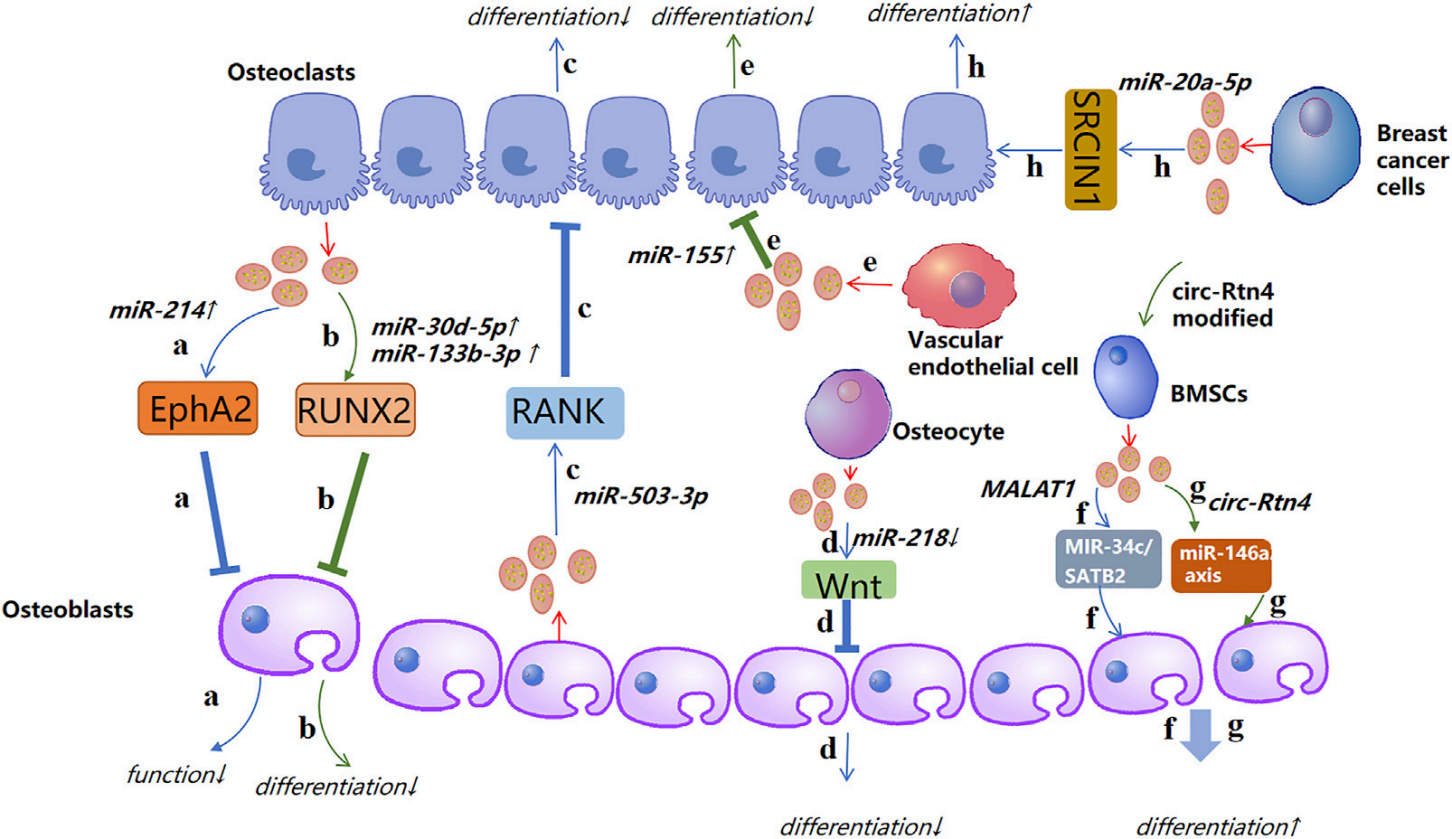
A possible mechanism of action of exosomal non-coding RNAs in osteoporosis (schematic). Different sources of exosomal non-coding RNAs, such as miRNA, lncRNA, circRNA, and tRNA, promote or inhibit the activity of osteoblasts and osteoclasts by regulating different targets, thereby affecting the course of osteoporosis. ↑: promotion; ↓: inhibition.

Yang et al. (2020a) revealed that exosomal miR-1263 derived from human umbilical cord mesenchymal stem cells (hUCMSCs) could inhibit BMSC apoptosis and prevent disuse OP in rats. Guo et al. (2019) showed that miR-20a-5p transported from breast cancer cell-derived exosomes promoted the proliferation and differentiation of osteoclasts by targeting the SRC kinase signaling inhibitor 1 gene. Song et al. (2019) confirmed that blocking the level of exosomal miR-155 secreted by vascular endothelial cells (EC) can reverse the inhibition of osteoclast differentiation of BMMS and further prevent bone resorption, indicating that exosomes miR-155 may have potential to treat osteoporosis. That was the first time that vascular endothelial cells had been found to treat OP. The low yield of exosomes hinders their clinical popularization. Therefore, Fan et al. (2020) suggested “exosome mimetics” as an alternative strategy to generate exosome-associated vesicles with high yields and enhanced regeneration capacity. Recently, Li et al. (2021) showed that that exosomal miR-186 isolated from BMSCs could promote the osteogenesis observed in postmenopausal OP through the hippo-signaling pathway. Hence, the therapeutic effect of exosomal miRNAs in OP has great potential (Figure 4).

The literature suggests that many miRNAs have high expression in exosomes, and that exosomal miRNAs can promote or inhibit osteogenesis by regulating cellular differentiation through signaling pathways or related proteins, which has huge implications for the clinical treatment of OP. In conclusion, exosomal miRNAs from different sources have important roles in the pathological process and treatment of OP. In addition, exosomal miRNAs may be clinical markers for OP diagnosis.

#### Roles of Exosomal miRNAs in OA

OA is a degenerative disease of the joints that causes chronic pain, cartilage degeneration, synovitis, disability, and carries an economic burden (Zarb and Carlsson, 1999; Loeser et al., 2012; Berenbaum, 2013; Glyn-Jones et al., 2015). ECM loss and cartilage destruction are the main features of OA (Kobayashi et al., 2005). As a weightbearing joint, the knee joint is a common site for OA. Because of the poor blood supply of cartilage and the weak potential of chondrocyte proliferation/migration, the regeneration ability of articular cartilage is very limited (Yuan et al., 2017). Clinical treatment of OA is aimed at alleviating pain symptoms. If OP progresses, it can be treated only with joint-replacement surgery, but complete repair and regeneration of damaged articular cartilage is not possible (Toh et al., 2017).

**FIGURE 5:**
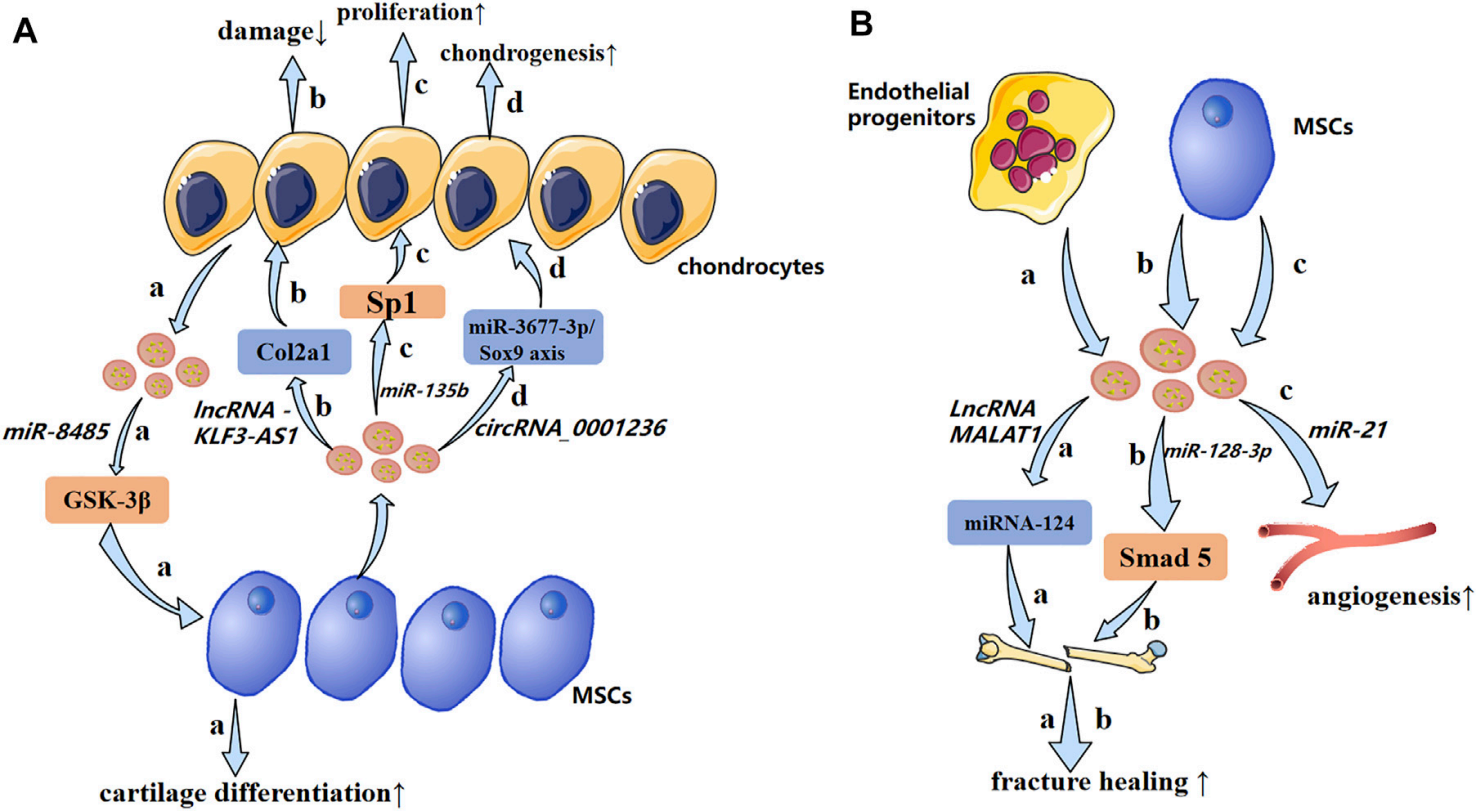
A possible mechanism of exosomal non-coding RNAs in osteoarthritis and impaired fracture healing (schematic). **(A)** Exosomal non-coding RNAs secreted by chondrocytes and MSCs produce pathological effects on each other through different mechanisms. **(B)** Endothelial progenitors and MSCs promote fracture healing and angiogenesis by transporting lncRNAs and miRNAs, respectively. ↑: promotion; ↓: inhibition.

In recent years, the role of exosomal miRNAs in OA has been studied (Figure 5A). For example, exosomes derived from hBMSCs overexpressing miR-26a-5p have been shown to delay damage to synovial fibroblasts *in vitro* and reduce OA damage *in vivo*. hBMSC-derived exosomes with high expression of miR-26a-5p in OA have been shown to inhibit expression of an inhibitor of synovial-fibroblast damage, prostaglandin-endoperoxide synthase 2, which is very important for OA treatment (Jin et al., 2020b). Sun et al. (2019) suggested that exosomes extracted from hBMSCs with high expression of miR-320c were better than exosomes extracted from control hBMSCs in terms of promoting the proliferation of hBMSC chondrocytes and downregulating matrix metallopeptidase (MMP)13 expression. Studies have shown that *TGF-β1* regulates Sp1through MSC-exosomal miR-135b to promote chondrocyte proliferation, thereby promoting cartilage repair (Wang et al., 2018). Mao et al. (2018b) established that miR-92a-3p expression was increased in the exosomes of MSC chondrocytes. Importantly, therapy using exosomal miR-92a-3p from MSCs can promote cartilage proliferation and expression of ECM genes in MSCs. Conversely, by enhancing expression of the Wnt family member 5a gene, therapy using exosomal miR-92a-3p from MSCs inhibits cartilage differentiation and reduces ECM synthesis in cartilage. Those results suggest that exosomal miRNAs derived from MSCs and hBMSCs are involved in regulation of the pathological process of OA.

Levels of exosomal miR-193b in the plasma of OA patients are lower than those in healthy people. miR-193b can target the histone deacetylase (HDAC)3 gene, thereby promoting acetylation of histone H3 and regulating the metabolism of primary human chondrocytes (Lin et al., 2014). Tao et al. (2017) revealed that exosomes derived from human synovial MSCs overexpressing miR-140-5p could promote cartilage regeneration and delay the progression of knee OA. miR-200C appears to have an important role in reducing interleukin (IL)-6-mediated inflammation. In addition, Withrow et al. used polymerase chain reactions (PCRs) to identify “exosomal cargoes.” Examination of exosomes in the synovial fluid of OA patients and non-OA patients showed that miR-200C expression was increased by 2.5-times in the exosomes of OA cases (Rokavec et al., 2012). Scholars have tested if miRNAs have expression differences in the exosomes of IL-1β-stimulated synovial fibroblasts compared with those of synovial fibroblasts of a control group. Fifty miRNAs had differential expression in the exosomes of synovial fibroblasts stimulated by IL-1β. Among them, miR-4454 was related to inflammatory stimulation, and miR-199b was related to cartilage formation (Zhang et al., 2012b; Zhou et al., 2014). Inflammatory factors may be links in stimulating exosomal ncRNAs to regulate OA. HDAC2/8 has been shown to inhibit cartilage development by inhibiting expression of cartilage-specific genes. Exosomes derived from miR-95-5p-overexpressing primary chondrocytes regulate cartilage development by targeting *HDAC2/8* directly (Mao et al., 2017; Mao et al., 2018a). Therefore, exosomal miRNAs may be a new direction for targeted therapy of OA.

High expression of miR-100-5p has been detected in stem cells from human exfoliated deciduous teeth-exosomes (SHED-Exos). miR-100-5p targets the 3′ untranslated region of mammalian target of rapamycin (mTOR) directly, and SHED-Exos miR-100-5p inhibits temporomandibular joint (TMJ) inflammation (Luo et al., 2019). miR-100-5p could be a new drug for treatment of TMJ inflammation. Liu et al. (2018a) suggested that expression of miRNAs in the exosomes of subchondral osteoblasts in patients with OA was significantly different compared with that of healthy people. Upregulated miR expression may be involved in the occurrence and progression of OA. Besides, it was worth noting that hsa-miR-4717-5p was the differentially expressed gene with the largest folding changes, which target gene was *RGS2*. Simultaneously, reverse transcription-quantitative polymerase chain reaction (RT-qPCR) showed that hsa-miR-4717-5p expression in the OA group was higher than that in the control group. Injection of BMSC-derived exosomal miR-9-5p in a mouse model of OA could reduce inflammation and OA-like injury. The main manifestations were downregulation of proinflammatory factors and a reduction in oxidative stress damage. Syndecan 1 was the target gene of miR-9-5p, and its upregulation led to exacerbation of inflammation and OA-like damage, contrary to that observed with exosomal miR-9-5p (Jin et al., 2020a). Exosome-like vesicles from chondrocytes of OA patients were shown to stimulate inflammasome activation and increase production of mature IL-1β by macrophages through the miR-449a-5p/ATG4B/autophagy pathway, thereby aggravating synovitis and accelerating OA progression (Ni et al., 2019). miR-8485 from the exosomes of chondrocytes could stimulate the cartilage differentiation of BMSCs by activating the Wnt/β-catenin pathway. This mechanism was related to inhibition of expression of the glycogen synthase kinase-3β gene by exosomes (Li et al., 2020). Wu et al. (2019) revealed that exosomes from infrapatellar fat-pad MSCs could promote the abnormal gait of OA mice and reduce the pathological changes of articular cartilage *in vivo*. RNA-sequencing of exosomes demonstrated that miR-100-5p had high expression in exosomes from infrapatellar fat-pad MSCs, and might regulate the biological behavior of chondrocytes by inhibiting the mTOR signaling pathway.

In summary, exosomal miRNAs from different sources are being discovered gradually, and may become a therapeutic method in OA (Table 2). Use of exosomal miRNAs as a means of treatment and diagnosis of OA may be an emerging direction for future clinical research.

**TABLE 2:** The role of exosomal non-coding RNAs in osteoarthritis.

Origin of exosomes	Exosomes cargo	Pathway	ncRNA expression	Mechanism	References
HBMSCs	miR-26a-5p	PTGS2	high	could delay synovial fibroblast damage *in vitro* and reduce OA damage	Jin et al. (2020b)
HBMSCs	miR-320c	N/A	high	promoting the proliferation of HBMSC chondrocytes and down-regulating matrix metallopeptidase 13	Sun et al. (2019)
MSCs	miR-135b	Sp1	high	promote chondrocyte proliferation, thereby promoting cartilage repair	Wang et al. (2018)
MSCs chondrocyte	miR-92a-3p	WNT5A	high	promoted cartilage proliferation and matrix gene expression in MSCs.	Mao et al. (2018b)
Plasma	miR-193b	HDAC3	low	promoting histone H3 acetylation and regulating the metabolism of primary human chondrocytes	Lin et al. (2014)
human synovial mesenchymal stem cells	miR-140-5p	N/A	high	promote cartilage regeneration and delay the progression of knee OA	Tao et al. (2017)
synovial fluid	miR-200C	N/A	high	miR-200C increased 2.5 times in OA exosomes compared to non-OA patients	Rokavec et al. (2012)
primary chondrocytes	miR-95-5p	HDAC2/8	high	regulated cartilage development and homogenous balance by directly targeting HDAC2/8	Mao et al. (2018a)
human exfoliated deciduous teeth	miR-100-5p	mTOR3’ untranslated region	high	inhibited the inflammation of temporomandibular joint (TMJ) chondrocytes	Luo et al. (2019)
subchondral osteoblasts	hsa-miR-4717-5p	RGS2	high	differentially expressed gene with the largest folding changes in the occurrence and progression of OA	Liu et al. (2018a)
BMSCs	miR-9-5p	SDC1	N/A	reduce inflammation and OA-like injury	Jin et al. (2020a)
chondrocytes	miR-8485	Wnt/β-catenin, GSK-3β	N/A	stimulating the cartilage differentiation of BMSCs	Li et al. (2020)
IPFP-MSCs	miR-100-5p	mTOR	high	promote the abnormal gait of OA mice and reduce the pathological changes of articular cartilage *in vivo*	Wu et al. (2019)
chondrocytes	lncRNA HULC	N/A	high	promoted cell apoptosis and inhibits cell proliferation	Song et al. (2017)
synovial fluid	lncRNA PCGEM1	N/A	high	the exosomal lncRNA PCGEM1 may be a novel indicator to distinguish early OA from late OA	Zhao and Xu, (2018)
MSC^KLF3-AS1^	lncRNA KLF3-AS1	miR-206/GIT1 axis	high	promoted the expression of GIT and alleviated the chondrocyte damage induced by IL-1β	Liu et al. (2018b)
MSCs	lncRNA KLF3-AS1	Col2a1	high	inhibited IL-1β-induced chondrocyte apoptosis	Liu et al. (2018c)
The Fibroblast-Like Synoviocyte	lncRNA H19	miR-106b-5p/TIMP2 axis	low	inhibited the degradation of the matrix in OA	Tan et al. (2020)
Human chondrocyte cell	circ_0001846	miR-149–5p/WNT5B axis	high	modulated IL-1β-induced chondrocyte cell damage	Zhu et al. (2021)
Human chondrocyte cell	circ-BRWD1	miR-1277/TRAF6 axis	high	contributed to osteoarthritis development	Guo et al. (2021)
MSCs	circRNA_0001236	miR-3677-3p/Sox9 axis	high	enhanced chondrogenesis and suppress cartilage degradation	Mao et al. (2021)

#### Roles of Exosomal miRNAs in Impaired Fracture Healing

A fracture can be complete or partial fracture of bone structure caused by an external force or accumulated strain (Claes et al., 2012; Einhorn and Gerstenfeld, 2015; Zhang et al., 2018a). About 10% of patients with a fracture have delayed healing. Long-term treatment can bring physical and psychological discomfort to patients, unnecessary financial burdens to patients and their families as well as poor quality of life (Komatsu and Warden, 2010; Gomez-Barrena et al., 2015). Fracture healing is a complex process. The activation, proliferation, and differentiation of local MSCs or progenitor cells are affected by specific growth factors and cytokine cascades (Murata et al., 2014).

Researchers have focused gradually on the effect of exosomal ncRNAs on fracture healing (Figure 5B). Liu et al. (2020) demonstrated that Hypo-Exos (exosomes derived from MSCs under hypoxia) promotes angiogenesis, proliferation, and migration by transporting exosomal miR-126, thereby accelerating fracture healing. Meanwhile knockout of hypoxia-inducible factor (HIF)-1α expression resulted in a significant reduction of MSC-derived exosomal miR-126, thereby eliminating the influence of exosomes derived from MSCs under hypoxia, and suggesting that hypoxic pretreatment mediated the production of exosomal miR-126 by activating *HIF-1α*. Exosomal miR-128-3p of MSCs in old rats can regulate bone formation and fracture healing by targeting *Smad 5*. For older people, a small synthetic RNA (exosomal miR-128-3P) may be a promising strategy for fracture healing (Xu et al., 2020b). Xiong et al. (2020) revealed that miR-5106 was significantly overexpressed in M2 macrophage-derived exosomes (M2D-Exos), whereas expression was decreased in M1 macrophage-derived exosomes (M1D-Exos). They suggested that exosomal miR-5106 could target salt-induced kinase (SIK2) and SIK3 genes directly to induce osteogenic differentiation of BMSCs. More importantly, local injections of miR-5106 agonists or M2D-Exos at fracture sites could accelerate healing *in vivo*. Studies have shown that MSC-derived promotion of fracture healing is related to exosomal miRNAs. Differentially expressed miRNAs such as miR-21, miR-4532, miR-125b-5p, and miR-338-3p may help to enhance bone formation and angiogenesis (Furuta et al., 2016) (Table 3).

**TABLE 3:** The role of exosomal non-coding RNAs in impaired fracture healing.

Origin of exosomes	Exosomes cargo	Pathway	ncRNA expression	Mechanism	References
MSCs	miR-126	HIF-1α	low	promoted fracture healing	Liu et al. (2020)
MSCs	miR-128-3p	Smad 5	N/A	regulated bone formation and fracture healing	Xu et al. (2020b)
M1D	miR-5106	SIK2	high	induced osteogenic differentiation of BMSCs	Xiong et al. (2020)
M2D	SIK3	low			
MSCs	miR-21	N/A	high	may help to enhance bone formation and angiogenesis	Furuta et al. (2016)
	miR-4532				
	miR-125b-5p				
	miR-338-3p				
Endothelial progenitors	LncRNA MALAT1	miRNA-124	N/A	stimulated the recruitment of osteoclast precursor cells and differentiation leading to bone repair	Cui et al. (2019)

Compared with the study of exosomal miRNAs in OP and OA, the study of exosomal miRNAs in fracture healing is less deep. Recent research on exosomal miRNAs in fractures has focused mainly on bone formation and angiogenesis.

### Roles of Exosomal lncRNAs in Bone-Related Diseases

lncRNAs are a family of transcripts containing >200 nucleotides that do not encode proteins (Costa, 2010; Moran et al., 2012; Geisler and Coller, 2013; Ulitsky and Bartel, 2013). Due to their different positions relative to protein-coding genes, they can be divided into five categories: antisense; long intergenic non-coding RNAs (lincRNAs); sense-overlapping; sense intronic; processed transcript (Quinn and Chang, 2016; Huynh et al., 2017). lncRNAs have been shown to be involved in nuclear structure and gene expression as regulatory factors during development. They are involved in regulation of the cell cycle, differentiation, transcription, and translation (Hu et al., 2012; Rinn and Chang, 2012; Joh et al., 2014; Quinodoz and Guttman, 2014; Vance and Ponting, 2014). lncRNAs are essential for bone formation. For example, targeted destruction of an lncRNA called HOX antisense intergenic RNA led to metacarpal deformities and allogeneic transformation of the spine. Furthermore, lncRNAs are regulators in the osteogenesis process of MSCs (Li et al., 2013b; Gu et al., 2017; Tye et al., 2018). It has been reported that lncRNAs are related to the progression of some diseases, and are being studied as new therapeutic targets (Gutschner and Diederichs, 2012; Li et al., 2013a; Wahlestedt, 2013; Hrdlickova et al., 2014).

#### Roles of Exosomal lncRNAs in OP

Compared with the research of exosomal miRNAs in OP, the research of exosomal lncRNAs in OP has not made much progress. Yang et al. (2019) demonstrated that a BMSC-derived gene in exosomes, metastasis-associated lung adenocarcinoma transcript (MALAT)1, promoted osteoblast activity. In addition, the *in vivo* experimental results of an ovariectomized mouse model showed that miR-34c reversed the effect of *MALAT1*, and that special AT-rich sequence-binding protein 2 reversed the effect of miR-34c in ovariectomized mice. Multiple myeloma is characterized by the reduced osteogenic potential of MSCs. Li et al. (2018) found that exosomal lncRNA *RUNX2-AS1* from myeloma cells could be delivered to MSCs, thereby inhibiting the osteogenicity of MSCs. Their results suggested that osteogenic differentiation from multiple myeloma cells to MSCs was through a unique exosomal lncRNA *RUNX2-AS1*/*RUNX2* pathway.

*RUNX2-AS1* in exosomal lncRNAs may be a potential therapeutic target for the bone injury caused by multiple myeloma. Xu et al. (2020a) showed that the lncRNAs of exosomes secreted by osteoclasts affect osteogenesis during granule-induced osteolysis. They demonstrated that the exosomes of RAW264.7 cells induced by titanium particles repressed the osteogenic differentiation of MC3T3-E1 cells. According to analyses from the Gene Ontology database, Kyoto Encyclopedia of Genes and Genomes database and verification by RT-qPCR, they identified two candidate lncRNAs: NONMMUT000375.2 and NONMMUT071578. These two lncRNAs regulated expression of four important genes related to osteoclast differentiation: B-cell lymphoma 2, Wnt11, TGFβ, and 3-phosphoinositide-dependent protein kinase-1. Exosomal lncRNA *H19* isolated from BMSCs has been shown to promote osteogenesis through the Angpt1/Tie2-NO signaling pathway in CBS heterozygous mice, thereby alleviating OP (Behera et al., 2021). Whether exosomal lncRNA *H19* can promote osteogenesis in normal mice may become a new research direction.

Recent research has shown that exosomal lncRNAs also regulate the differentiation direction of cells through related proteins and signaling pathways, thereby affecting osteogenesis. Study of exosomal lncRNAs in bone-related diseases is an emerging research direction, but faces practical problems, such as the screening and isolation of exosomal lncRNAs.

#### Roles of Exosomal lncRNAs in OA

Scholars have hypothesized that selective packaging of ncRNA into exosomes could reflect the cellular response to cartilage-cell death during OA pathogenesis. Song et al. (2017) showed that expression of exosomal hepatocellular carcinoma upregulated long non-coding RNA (HULC) in OA patients was downregulated significantly, whereas exosomal miR-372-3p expression in OA patients was upregulated significantly. Besides, *HULC* overexpression in normal chondrocytes significantly promoted the apoptosis and inhibited the proliferation of cells. Zhao and Xu (2018) suggested that in synovial-fluid samples, exosome expression in the control group was significantly lower than that in patients with early OA or late OA. Furthermore, expression of the exosomal lncRNA prostate cancer gene expression marker (PCGEM)1 gene in late OA was significantly higher than that in early OA. And the early expression of OA was significantly higher than that of the control group. Therefore, they concluded that exosomal lncRNA *PCGEM1* may be a novel indicator to distinguish early OA from late OA.

Exosomes derived from KLF3-AS1-overexpressing-MSCs (MSC^KLF3-AS1^-Exos) have been shown to participate in MSC-Exos-mediated induction of chondrocyte proliferation *via* the miR-206/G-protein-coupled receptor kinase interacting protein (GIT)1 axis. *KLF3-AS1* promoted GIT1 expression by “sponging” miR-206 as a competitive endogenous RNA. In addition, MSC^KLF3-AS1^-Exos alleviated the chondrocyte damage induced by IL-1β (Zhang et al., 2015; Liu et al., 2018b). Liu et al. (2018c) demonstrated that lncRNA *KLF3-AS1* expression was upregulated significantly in MSC-Exos, and that exosomal *KLF3-AS1* inhibited IL-1β-induced chondrocyte apoptosis by upregulating expression of Col2a1 and aggrecan, and downregulating expression of *MMP13* and *Runx2*. Exosomal *KLF3-AS1* could also promote cartilage repair in a rat model of OA. Tan et al. (2020) reported that exosomal lncRNA *H19* may be a therapeutic target for OA because it promotes the proliferation and migration of chondrocytes by targeting the miR-106b-5p/tissue inhibitor of metalloproteinases 2 axis and inhibits ECM degradation in OA.

#### Roles of Exosomal lncRNAs in Impaired Fracture Healing

Only one study has been done on the role of exosomal lncRNAs in impaired fracture healing. The reason why few studies on exosomal lncRNAs in bone-related diseases have been done may be that fewer lncRNAs are carried in exosomes, or that lncRNAs have only slight effects on bone-related diseases.

Bone repair involves two main features: 1) bone resorption caused by osteoclastogenesis; 2) endothelial progenitor cells stimulate neovascularization and osteogenic differentiation. Cui et al. (2019) showed that endothelial progenitor cell-derived exosomal *MALAT1* was bound directly to miR-124. This action reduced inhibition of integrin subunit *β1* and promoted the migration and osteoclast differentiation of bone marrow-derived macrophages. This phenomenon stimulated the recruitment of osteoclast precursor cells and differentiation, which led to bone repair.

### Roles of Other Exosomal lncRNAs in Bone-Related Diseases

#### Roles of Exosomal circRNAs in OP

circRNAs are endogenous RNAs with covalent closed-loop structures. Neither 5–3 ends nor poly (A) tails are present mainly in cytoplasm. They are produced during RNA splicing and are generated by exons, introns or a combination of both (Owens et al., 2012; Li et al., 2015; Lasda and Parker, 2016). circRNAs are regarded as significant regulators of cell function, rather than a non-functional byproduct of abnormal RNA splicing (Salzman, 2016). Yang et al. (2020b) reviewed the development of circRNAs in OP. They showed that circRNAs act by targeting the major genes and signaling pathways related to osteoblast differentiation and osteoclast differentiation. For example, circRNA_0016624 has been reported to activate miR-98 and enhance expression of *BMP2* (which plays an important part in induction of osteogenic differentiation), so circRNA_0016624 promoted osteoblast differentiation. However, the role of exosomal circRNAs in OP has not been explored thoroughly.

Cao et al. (2020) demonstrated that co-cultivation with exosomes derived from circ-Rtn4 modified BMSCs (Rtn4-Exos) could reduce the cytotoxicity and apoptosis of mouse cells (MC3T3-E1) induced by the tumor necrosis factor (TNF)-α gene, which was manifested by expression of *caspase-3*, *caspase-3*, and *Bax* protein as well as a reduction in *caspase-3* activity. In particular, Rtn4-Exos exhibited its function in TNF-α-treated MC3T3-E1 cells by sponging miR-146a, which is the target of circ-RTN4. Thus, their findings indicated that Rtn4-Exos suppressed the cytotoxicity and apoptosis of MC3T3-E1 cells induced by *TNF-α* through sponging of miR-146a. Hence, Rtn4-Exos could be considered as a new drug candidate for OP treatment. Exosomal hsa_circ_0006859 isolated from the serum of postmenopausal women has been revealed to be a potential biomarker of postmenopausal OP. It enhances the adipogenic and osteogenic differentiation of hBMSCs by sponging miR-431-5p (Zhi et al., 2021).

Few studies have been carried out on exosomal circRNAs. However, exosomal circRNAs seem to influence the fate and differentiation of target cells through sponging miRNAs.

#### Roles of Exosomal circRNAs in OA

Zhu et al. (2021) reported that circ_0001846 has high expression in the exosomes of an IL-1β-treated human chondrocyte cell line (CHON-001). They showed that transfer of exosomal circ_0001846 regulates IL-1β -induced chondrocyte damage through the miR-149-5p/Wnt5B axis. Guo et al. (2021) demonstrated that exosomal circ-BRWD1 isolated from CHON-001 cells can affect the progression of CHON-001 cells induced by IL-1β by regulating the miR-1277/TRAF6 axis, thereby promoting OA development. Mao et al. (2021) showed that exosomal circRNA_0001236 transported by MSCs promotes expression of cartilage-specific genes and proteins *via* the miR-3677-3p/SRY-box transcription factor (SOX) 9 axis. Thus, exosomes overexpressing circRNA_0001236 mitigate cartilage degradation, inhibit osteoarthritis progression, and enhance cartilage repair.

Those results suggest the mechanism of action of exosomal circRNAs in OA. They provide a new direction for the study of exosomal circRNAs in OA and other bone-related diseases.

#### Roles of Exosomal tRNAs in OP

The nucleotide information on the mRNA is associated with the amino acid sequence tRNA by decoding the nucleotide triad to help the ribosome synthesize proteins. Besides this routine function, tRNAs are involved in the signal transduction, survival, and apoptosis of cells, metabolism of amino acids and porphyrins and stress responses (Giege, 2008; Phizicky and Hopper, 2010; Raina and Ibba, 2014). Recent studies have shown that tRNA-derived fragments (tRFs) are involved in post-transcriptional regulation and could be considered as therapeutic targets for certain diseases (Kumar et al., 2016).

The tRFs derived from tRNA or pre-tRNA are lncRNAs (Zhang et al., 2019a). Zhang et al. (2018b) found that, compared with those in healthy controls, there were 11 upregulated tRFs and 18 downregulated tRFs in exosomes in the plasma of OP patients. Among them, expression of exosomal tRF-25-R9ODMJ6B26 (tRF-25), tRF-38-QB1MK8YUBS68BFD2 (tRF-38) and tRF-18-BS68BFD2 (tRF-18) was increased significantly. In addition, expression of tRF-25, tRF-38, and tRF-18 in the plasma of OP patients had good accuracy for OP diagnosis, and could be used as diagnostic biomarkers for OP.

In addition to the exosomal ncRNAs reviewed above, exosomal mRNAs have a role in OP. Seven genes associated with mRNAs have been detected in the exosomes of differentiated hBMSCs: *ACIN1*, *DDX6*, *DGKA*, *DKK2*, *Lsm2*, *RPS2,* and *Xsox17*. They showed obvious differential expression and could induce differentiation into mineralized bone cells (Xu et al., 2014).

## Clinical Potential of Exosomal ncRNAs in Bone-Related Diseases

Sun et al. (2016) incubated exosomes isolated from OP patients and from the serum of healthy controls with osteoblasts. They showed that miR-214 expression was much higher upon incubation with exosomes from hFOB1.19 cells than that in non-OP individuals. Simultaneously, mRNA expression also decreased in osteogenesis-related genes. Those results suggest the clinical importance of increased miR-214 expression in the serum of OP patients, and suggest its potential use as a marker for the diagnosis and therapy of OP. Gao et al. (2019) reported that exosomal miR-320 isolated from leukemia cells could be taken up by BMSCs and bind to heterogeneous nuclear ribonucleoprotein A1 to inhibit osteogenic differentiation. Hence, heterogeneous nuclear ribonucleoprotein A1-mediated transfer of exosomal miR-320 from leukemia cells to BMSCs could be an important mediator of leukemia progression and a potential therapeutic target for chronic myelogenous leukemia.

Expression of exosomal lncRNA plasmacytoma variant translocation-1 in the serum of OA patients has been shown by Meng et al. (2020) to reduce progression of lipopolysaccharide-induced OA by mediating the high-mobility group protein 1/toll-like receptor 4/nuclear factor-kappa B pathway through miR-935p. Liang et al. (2020) reported that chondrocyte-affinity peptides in exosomes reduced OA progression in a rat model by delivering miR-140 to the deep cartilage area and inhibiting cartilage degradation, which points to organelle-based acellular OA treatment.

Apart from OP, OA, and bone fractures, we also found meaningful clinical studies of exosomal ncRNAs in other bone-related diseases. For example, Fang et al. (2020) demonstrated that, compared with healthy individuals, expression of tRNA-derived small RNA (tsRNA)-10277 in the exosomes of plasma of patients with steroid-induced osteonecrosis of the femoral head (SONFH) was downregulated significantly. Also, exosomes in BMSCs loaded with tsRNA-10277 enhanced the osteogenic differentiation of dexamethasone-induced BMSCs. That study provides new ideas for the osteogenic effects of exosomes in BMSCs carrying specific tsRNAs on SONFH. Besides, there is evidence that exosomal lncRNA nuclear enriched abundant transcript (NEAT)1 isolated from the serum of patients with rheumatoid arthritis can promote the development of rheumatoid arthritis through the miR-144-3p/ROCK2 axis (Liu et al., 2021). This *NEAT1/*miR-144–3p/ROCK2 regulatory pathway may become a new treatment target for rheumatoid arthritis.

## Future Perspectives

Bone-related diseases not only affect quality of life, but also threaten life in severe cases. Accumulating evidence suggests that ncRNAs with regulatory effects are essential for the pathogenesis of bone-related diseases.

From the research of exosomes and ncRNAs, some common points can be found in the research of bone-related diseases. For example, exosomal miR-155 can inhibit osteoclast activity (Song et al., 2019) and miR-155 expression is upregulated significantly in osteoclasts (Zhang et al., 2012a; Zhao et al., 2017). In addition, reduced expression of miR-155 can target the gene for the leptin receptor and increase its expression through the 5′ adenosine monophosphate-activated protein kinase signaling pathway which, ultimately, represses osteoclast activation and bone resorption of osteoclasts in alendronate-treated osteoporotic mice (Mao et al., 2019). This commonality is not accidental, and merits in-depth exploration. Liu et al. (2015a) demonstrated that BMSC transplantation can rescue bone loss in Fas-deficient MRL/LPR mice by secreting exosomes. This is achieved by reducing miR-29b expression, thereby enhancing osteogenic differentiation *in vitro* and promoting bone formation *in vivo*. Petho et al. (2018) reported that miR-677-3p, miR-680, miR-3084-3p and miR-5000 had high expression in the exosomes of mineralized osteoblasts. Furthermore, Kumar et al. (2017) reviewed application of miRNAs as peripheral biomarkers in aging and age-related diseases, including OP.

Research on exosomal ncRNAs and OA has also shown a certain connection. The chondrocytes were treated with channel protein connexin 43 (Cx43) exosomes released by OA chondrocytes, osteocytes and synovial cells eventually increased cell senescence levels and senescence-associated secretory phenotypes through p53/p16 and NF-kß (Varela-Eirín et al., 2019). In general, lncRNA *PCGEM1* in the synoviocytes of OA patients targets miR-770 to stimulate synoviocyte proliferation by acting as a spongy lncRNA (Kang et al., 2016). M2 macrophages polarized by lncRNA *MM2P* significantly strengthen chondrocyte function and promote the delivery of M2-derived exosomal SOX9 to chondrocytes (Bai et al., 2020). Future studies can explore if these lncRNAs are carried in exosomes and are involved in OA regulation. Zavatti et al. (2020) revealed the efficacy of exosomes in human amniotic-fluid SCs against cartilage damage, indicating a positive correlation with their *TGF-β* content. circRNAs are relatively uncharted territory in OA. Zhou et al. (2019b) established that, through sponging miR-127-5p, circRNA.33186 encouraged OA pathogenesis. MSC- and BMSC-derived exosomes make a great contribution to fracture healing, but the mechanism of action needs further exploration (Furuta et al., 2016; Hao et al., 2017). Besides, hUCMSC-derived exosomes can repair bone fractures in rats mainly through the Wnt signaling pathway (Zhou et al., 2019a). Most of the exosomes that can be used to treat bone-related diseases come from SCs. Hao et al. (2017) reviewed a promising strategy for SC-derived exosomes to heal fractures. However, Borel et al. (2020) found that prostate cancer-derived exosomes could promote the differentiation and activity of osteoblasts through the phospholipase-D2 pathway. That discovery broadens investigation of the mechanism of action of exosomes in bone-related diseases. Also, treatment of rheumatoid arthritis with exosomal ncRNAs shows great prospects for development (Ding et al., 2020; Meng and Qiu, 2020).

Although studies have shown that exosomal ncRNAs are emerging diagnostic markers and disease targets, the transformation from basic science to clinical application will be challenging. Looking for commonality between bone-related diseases may aid their treatment using exosomes. Certainly, investigation of the connection points between miR-155 in exosomes and osteoclasts could yield interesting results.

## Conclusion

Study of exosomal ncRNAs in OP is more in-depth than that in OA and impaired fracture healing. As important cell communicators, exosomes (and their contents) have indispensable roles in the occurrence, development, and treatment of OP, OA, and fracture. Exploring the detailed mechanism of action of exosomal ncRNAs in bone-related diseases will help transformation from basic research to their clinical application in bone-related diseases.

## Abbreviations

OP, osteoporosis; OA, osteoarthritis; BMSCs, bone marrow mesenchymal stem cells; lncRNA, long non-codingRNA; miRNA, microRNA; tRNAs, transfer RNA; circRNAs, circular RNA; hBMSCs, human bone marrow mesenchymal stem cells; hUCMSCs, human umbilical cord mesenchymal stem cells; ECM, extracellular Matrix; SHED, human exfoliated deciduous teeth; TMJ, temporomandibular joint; SIK2, salt-induced kinase 2 and 3; GIT1, G-protein-coupled receptor kinase interacting protein 1.
